# Genetic Adjuvants in Replicating Single-Cycle Adenovirus Vectors Amplify Systemic and Mucosal Immune Responses against HIV-1 Envelope

**DOI:** 10.3390/vaccines8010064

**Published:** 2020-02-02

**Authors:** William E. Matchett, Goda Baddage Rakitha Malewana, Haley Mudrick, Michael J. Medlyn, Michael A. Barry

**Affiliations:** 1 Virology and Gene Therapy (VGT) Graduate Program, Mayo Clinic, Rochester, MN 55905, USA; matchett.william@mayo.edu; 2 Mayo Summer Undergraduate Research Fellow (SURF), Mayo Clinic, Rochester, MN 55905, USA; malewanag@husson.edu; 3 Molecular Pharmacology and Experimental Therapeutics (MPET) Graduate Program, Mayo Clinic, Rochester, MN 55905, USA; mudrick.haley@mayo.edu; 4 Immunology Graduate Program, Mayo Clinic, Rochester, MN 55905, USA; medlyn.michael@mayo.edu; 5 Department of Internal Medicine, Division of Infectious Diseases, Mayo Clinic, Rochester, MN 55905, USA; 6 Department of Immunology, Mayo Clinic, Rochester, MN 55905, USA; 7 Department of Molecular Medicine, Mayo Clinic, Rochester, MN 55905, USA

**Keywords:** HIV-1, single-cycle adenovirus, gene-based vaccines, genetic adjuvants

## Abstract

Most infections occur at mucosal surfaces. Providing a barrier of protection at these surfaces may be a useful strategy to combat the earliest events in infection when there are relatively few pathogens to address. The majority of vaccines are delivered systemically by the intramuscular (IM) route. While IM vaccination can drive mucosal immune responses, mucosal immunization at intranasal (IN) or oral sites can lead to better immune responses at mucosal sites of viral entry. In macaques, IN immunization with replicating single-cycle adenovirus (SC-Ads) and protein boosts generated favorable mucosal immune responses. However, there was an apparent “distance effect” in generating mucosal immune responses. IN immunization generated antibodies against HIV envelope (env) nearby in the saliva, but weaker responses in samples collected from the distant vaginal samples. To improve on this, we tested here if SC-Ads expressing genetic adjuvants could be used to amplify antibody responses in distant vaginal samples when they are codelivered with SC-Ads expressing clade C HIV env immunogen. SC-Ads env 1157 was coadministered with SC-Ads expressing 4-1BBL, granulocyte macrophage colony-stimulating factor (GMCSF), IL-21, or *Clostridoides difficile (C. diff.)* toxin fragments by IN or IM routes. These data show that vaginal antibody responses were markedly amplified after a single immunization by the IN or IM routes, with SC-Ad expressing HIV env if this vaccine is complemented with SC-Ads expressing genetic adjuvants. Furthermore, the site and combination of adjuvants appear to “tune” these antibody responses towards an IgA or IgG isotype bias. Boosting these priming SC-Ad responses with another SC-Ad or with SOSIP native-like env proteins markedly amplifies env antibody levels in vaginal washes. Together, this data may be useful in informing the choice of route of delivery adenovirus and peptide vaccines against HIV-1.

## 1. Introduction

Up to 90% of HIV-1 infections occur at mucosal surfaces after sexual contact [1]. It is thought that only one or a few virions infect the host during these exposures [2]. Given the lower number of viruses, blocking these first infection events may be useful to halt HIV infections [2]. Many believe that HIV vaccines can be delivered by the intramuscular (IM) route to generate adequate mucosal immune responses to provide this barrier protection [3,4,5,6]. Replication-defective adenovirus (RD-Ad) vaccines can protect against simian/human immunodeficiency virus (SHIV) and SIVmac251 after IM immunization in rhesus macaques, particularly when used in combination with protein boosts [7,8]. These and other data led to the APPROACH (NCT02315703), TRAVERSE (NCT02788045), ASCENT (NCT02935686), and the ongoing Mosaico (NCT03964415) human vaccine trials [9,10]. The Mosacio trial uses RD-Ad26 to deliver a combination of HIV immunogens followed by gp140 protein boosts.

**Figure 1:**
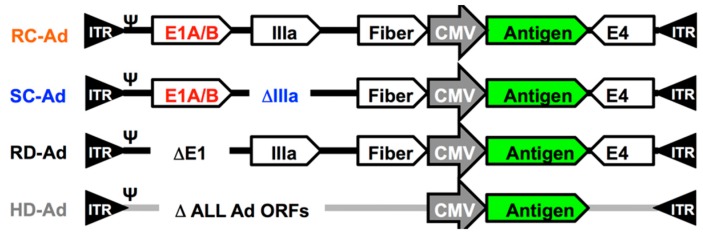
Adenovirus vaccines. Key Ad and adenovirus genes relevant to the vaccine functions are shown. Replication-competent RC-, single-cycle SC-, and replication-defective (RD)-Ads all carry most Ad open reading frames (ORFs) (not shown). HD-Ads are deleted for all of these adenovirus ORFs.

The vaccines used in the aforementioned studies are E1-deleted RD-Ads (Figure 1). An E1-deleted Ad infects a cell, delivers its one copy of an HIV antigen gene, and expresses “1X” of these antigens. They are safe, but do not replicate transgenes or their expression. In contrast, an E1-intact replication-competent Ad (RC-Ad) (Figure 1) infects the cell, but replicates and amplifies the same antigen gene 10,000-fold in the cell. In so doing, an RC-Ad produces 100s of times more antigen per cell and provokes significantly stronger immune responses than RD-Ad vectors [11,12,13,14,15,16,17,18,19,20,21,22,23,24,25,26,27,28,29,30,31,32,33,34,35,36,37,38].

While RC-Ads are documented to be more potent than RD-Ad vectors, replication-competent Ads pose a real risk of causing adenovirus infections as a side-effect of vaccination. This risk is significant enough that when live RC-Ad vaccines are used in military recruits, they are encapsulated and swallowed to prevent causing Ad respiratory infections [39]. RC-Ad4 influenza vaccines have more recently been used to provide long lasting antibody responses after a single intranasal (IN), tonsillar, or oral immunization in phase I trial NCT01443936 [40]. While these RC-Ad vaccines provided potent immune responses, 63% of IN vaccinees developed symptoms of respiratory infection due to the viral vaccine (Dr. Mark Connors, NIH, personal communication). These data suggest that replicating Ad vaccines can be potent, but that there are significant risks of adenovirus infections.

We developed single-cycle Ad (SC-Ad) vectors to avoid the risks of adenovirus infections associated with RC-Ads, but allowed for antigen gene replication. SC-Ad vectors retain their E1 genes to allow DNA replication, but are deleted for their pIIIA capsid gene to block the production of infectious Ad progeny virions [37,38,41,42] (Figure 1).

SC-Ads replicate their genomes and transgenes up to 10,000-fold like RC-Ads [37]. RC- and SC-Ad produce up to 300-fold higher protein than RD-Ad [37]. SC-Ads generate more robust and more persistent immune responses than either RD-Ad or RC-Ads [38]. For example, after single intranasal immunization, only SC-Ad generated antibodies in vaginal washes that rose over 6 months (RD and RC did not). RC-Ad induces stronger antiviral interferon stimulated gene (ISG) responses than SC-Ad [42]. This or other factors may blunt RC-Ad vaccine efficacy relative to SC-Ad.

SC-Ads generate antibodies and T cells responses that increase over 12 months after single immunization vs. HIV, influenza, Ebola, Zika, or *C. difficile* antigens [38,41,42,43,44,45,46,47,48]. SC-Ad carrying influenza hemagglutinin (HA) produced markedly more antigen than RD-Ad in vitro, requiring 33-fold less virus to produce the same amount of HA [41]). In vivo, SC-Ad produced significantly higher anti-influenza hemagglutination inhibition (HAI) antibodies than RD-Ad and provided better protection against intranasal influenza challenge in cotton rats after single immunization [41]. An SC-Ad vaccine expressing Ebola glycoprotein (gp) protected against pseudo-challenge with vesicular stomatitis virus (VSV) pseudotyped with Ebola gp a year and a half after single immunization in hamsters [47]. This SC-Ad generated anti-Ebola antibody responses with similar kinetics and levels as were generated by replication-competent VSV-EBOV-Luciferase vector [47]. This is notable, since SC-Ad does not replicate in mice, whereas VSV-EBOV is replication-competent. We more recently used the SC-Ad platform to vaccinate against the bacterial pathogen *Clostridoides difficile (C. diff.).* SC-Ad expressing the receptor-binding domains of *C. diff.* toxin A and B (TcdA/B) protected animals from lethal challenges more than 38 weeks after a single immunization [45].

SC-Ad serotype 6 vectors expressing HIV clade B envelope sequences were used to vaccinate rhesus macaques by the IN or IM [43]. Single immunization by the IM route generated significant envelope antibodies within four weeks. Each SC-Ad6-primed group was boosted twice by either the IM or the IN route with SC-Ad6 and SC-Ad657 vectors. Endpoint and midpoint titers showed that these SC-Ad prime-boosts generated increasing envelope antibodies in all groups except in animals that were immunized only by the IN route. Most HIV Ad vaccines are amplified with protein boosts. All SC-Ad-env groups were boosted with recombinant gp140 protein. These protein boosts increased midpoint binding titers by two orders of magnitude in all of the groups. Interestingly, the IN-IN-IN group, which had no antibodies at week 24, boosted as strongly as the other groups [43]. These immunizations generated significant cellular responses and antibody-dependent cellular cytotoxicity (ADCC) activity and clade B HIV neutralizing antibodies [43].

While these results were significant, these studies also revealed a weakness in applying SC-Ad vaccines by the mucosal IN vaccine route. Final saliva and vaginal samples from the animals had detectable envelope binding IgG antibodies in all groups. However, there was a distance effect on these antibodies. Animals that were immunized predominantly by the mucosal route had env-binding antibodies in their saliva near the site of immunization. However, only a few of these animals had antibodies at the more distant vaginal site [43].

These data suggest that there is value in mucosal vaccination, but that responses that are generated by immunization at an “easy” mucosal site, like the nose, may not effectively transmit to distant vaginal and rectal mucosal barriers that are relevant to HIV infection.

Given this putative distance effect, we here tested if coimmunization with genes encoding genetic adjuvants might be able to reduce this problem.

After plasmid DNA vaccines or gene-based vaccines were initially developed, there were soon efforts to try to amplify the level of immune responses by codelivery of plasmids expressing genes like granulocyte macrophage colony-stimulating factor (GMCSF) [49,50], B7 [51], IL-10, and IL-12 [52], and many others. Some of the earliest examples of protection against HIV or SHIV in nonhuman primates was observed with coimmunization of genetic adjuvants [53,54].

Genetic adjuvants have usually been used by the IM route to amplify systemic immune responses, not to improve mucosal responses. In this study, SC-Ad vectors expressing a clade C envelope antigen were coimmunized with SC-Ads expressing 4-1BBL, GMCSF, IL-21, and a novel mucosal adjuvant that expresses the receptor-binding domains of *C. diff.* toxin A and B (TcdA/B) [45]. We tested if SC-Ads expressing genetic adjuvants could amplify mucosal responses and reduce the mucosal distance effect.

## 2. Materials and Methods

### 2.1. Single-Cycle Adenovirus Vectors

A clade C gp140 envelope from SHIV-1157ipd3N4 [55] was codon-optimized and synthesized by Genscript. This cDNA was inserted into a cytomegalovirus promoter and SV40 poly cassette as in reference [37]. This cassette was recombined in between the fiber and E4 genes of SC-Ad6, and the virus was rescued as in reference [37]. The virus was amplified in 293-IIIA cells and purified on two CsCl gradients. Viral particles (vp) were quantified by OD260. The cDNAs for mouse GMCSF, 4-1BBL, and IL-21 were inserted and rescued by the same methods. A codon-optimized *C. difficile* TcdA/B gene was synthesized as described in reference [45], was inserted, and this SC-Ad6 was rescued as described above.

### 2.2. SOSIP Protein Vaccine

Native-like stabilize env SOSIP trimers [56,57,58] were used for protein boosts. SOSIP proteins contain a disulfide link between residues 501 and 605 and an Ile-to-Pro mutation at residue 559. Clade C CZA97 SOSIP.v4.2-M6.IT produced from CHO cells was generously supplied by Dr. John Moore. Five micrograms SOSIP protein was mixed with the NKT cell adjuvant alphaGalCer that we had used previously [59,60].

### 2.3. Animals

All animal handling and experiments were carried out according to the provisions of the Animal Welfare Act, PHS Animal Welfare Policy, the principles of the NIH Guide for the Care and Use of Laboratory Animals, and the policies and procedures of the Institutional Animal Care and Use Committee at Mayo Clinic. Mice were purchased from Charles River Laboratories. The mice were housed in the Mayo Clinic Animal Facility.

### 2.4. Immunizations and Sample Collection

Mice were anesthetized with isoflurane and immunized by the IN or IM route with the indicated amounts of the indicated SC-Ad vectors. The mice were anesthetized, blood was collected from their facial vein, and vaginal washes were collected at the indicated time points.

### 2.5. Enzyme-Linked Immunosorbent Assay (ELISA)

ELISAs were performed with CN54 clade C gp140 protein from NIH AIDS Reagent Program. The antigen was diluted in phosphate-buffered saline (PBS) and incubated overnight in Immulon 4 HBX plates (Thermo) at 100 ng/well. The wells were blocked with 5% milk in Tris-buffered saline with 0.1% Tween 20 (TBST) at room temperature (RT) for 2 h. The indicated dilutions of each sample were plated in triplicate and incubated for 3 h at RT. The wells were washed, and goat anti-mouse IgG or IgA horseradish peroxidase (Thermo Fisher Scientific Inc.) was added and incubated 2 h at RT. Wells were washed and 1 step Ultra TMB ELISA (Thermo Fisher Scientific Inc.) was added to each well. Color development was terminated by the addition of H_2_SO_4_. OD450 was determined on a plate reader.

### 2.6. Sub-Isotyping Enzyme-Linked Immunosorbent Assay (ELISA)

ELISA plates were prepared as above with CN54 clade C gp140 protein from NIH AIDS Reagent Program. After the incubation of the primary antibody at the indicated dilution, the wells were washed and rabbit anti-mouse IgM, IgA, IgG1, IgG2A, IgG2B, or IgG3 antibodies were added and allowed to incubate for 2 h at RT. Wells were washed, and goat anti-rabbit IgG peroxidase (MilliporeSigma) was added to each well and incubated for 1 h at RT. Wells were washed and 1 step Ultra TMB ELISA was added to all the wells. The reaction was stopped by the addition of H_2_SO_4_, and the plates were read at OD450 on a plate reader.

### 2.7. Data Analysis

Statistical analyses were performed using Prism Graphical software.

## 3. Results

### 3.1. SC-Ad6 Expressing Clade C HIV Envelope and Genetic Adjuvants

**Figure 2:**
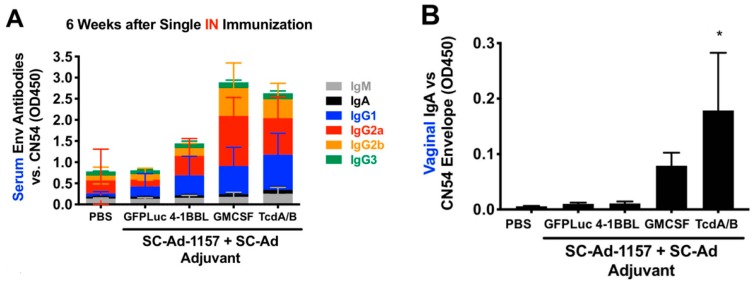
Effects of SC-Ad genetic adjuvants on clade C env antibody responses in mice after intranasal (IN) immunization. Groups of 10 female BALB/c mice were immunized with phosphate-buffered saline (PBS) or 10^9^ vp of the indicated SC-Ads. Six weeks later, samples were collected for enzyme-linked immunosorbent assay (ELISA) vs. clade C CN54 gp140. (**A**) Sub-isotyping ELISA for the indicated samples at 1/200 dilution (low dilution used for low-sensitivity sub-isotyping kit). All IgG isotypes in the granulocyte macrophage colony-stimulating factor (GMCSF) and TcdA/B groups were significantly different than PBS by 2-way ANOVA. (**B**) ELISA OD450 levels are shown for 1/35 dilution of vaginal wash samples with detection by anti-IgA. * *p* < 0.05.

10^9^ viral particles (vp) of SC-Ad6 expressing clade C gp140 from SHIV-1157ipd3N4 (Figure 4E) was used to immunize BALB/c mice by the IN route in combination with 10^9^ SC-Ads expressing 4-1BBL, GMCSF, *C. diff* toxin fragment TcdA/B, or a nonspecific adenovirus control expressing GFP-Luciferase (Figure 2). *C. diff* TcdA/B was included, since others have shown that bacterial toxins can be potent mucosal adjuvants [61,62,63].

ELISAs using serum collected 6 weeks after single immunization demonstrated significant increases in antibody isotypes by GMCSF and TcdA/B (Figure 2A, *p* < 0.05 or less for all IgGs). When vaginal washes were assayed for IgA at the same time point, this revealed similar trends, with highest mucosal IgA mediated by IN codelivery of TcdA/B adjuvant (Figure 2B).

**Figure 3:**
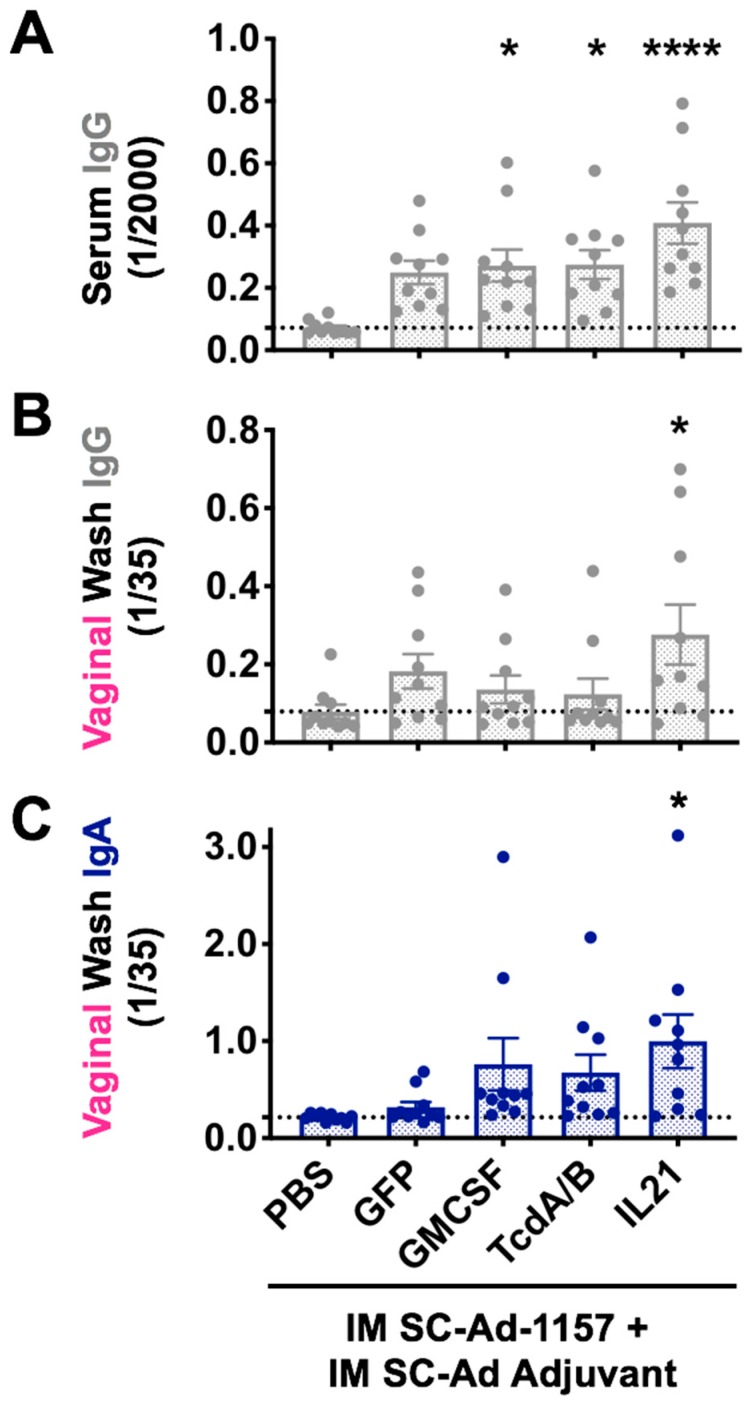
Effects of SC-Ad genetic adjuvants on clade C env antibody responses in mice after intramuscular (IM) immunization. Groups of 10 female BALB/c mice were immunized with PBS or 10^10^ vp of the indicated SC-Ads. Six weeks later, samples were collected for ELISA vs. clade C CN54 gp140 (**A**)**,** (**B**), and (**C**) six week ELISAs after a single high dose IM immunization. Mean +/− SEM is shown. (**A**) 1/2000 sera dilutions detecting IgG. (**B**) IgG ELISA for 1/35 dilution of vaginal wash samples. (**C**) IgA ELISA for 1/35 dilution of vaginal wash samples. * *p* < 0.05, **** *p* < 0.001 by one-way ANOVA.

To maximize antibody responses, SC-Ad-GMCSF and TcdA/B were tested again by the IM route with 10-fold more SC-Ad. We also added SC-Ad-IL-21 adjuvant for its ability to stimulate Tfh and other T cells. In this case, IM injections were administered to the quadricep muscles near to the vaginal sample site. Six weeks after this single higher dose IM immunization, ELISA with 1/2000 dilutions of sera showed increased env IgG levels by SC-Ad-GMCSF, TcdA/B, and IL-21 (Figure 3A, *p* < 0.05 vs. PBS). Notably, SC-Ad-IL-21 provided even higher antibody levels than GMCSF or TcdA/B (*p* < 0.0001 vs. PBS).

When vaginal wash samples were tested for IgG, all SC-Ad-1157 animals had increases, but only SC-Ad-IL-21 adjuvant reached significance (Figure 3B, *p* < 0.05 vs. PBS). When vaginal washes were assayed for IgA at the same time point, this revealed similar trends with higher mucosal IgA in most animals in the GMCSF, TcdA/B, and IL-21 groups; only the IL-21 group reached *p* < 0.05 (Figure 3C).

### 3.2. IM or IN Clade C SOSIP Protein Boost of SC-Ad-Env + SC-Ad-Adjuvants

**Figure 4:**
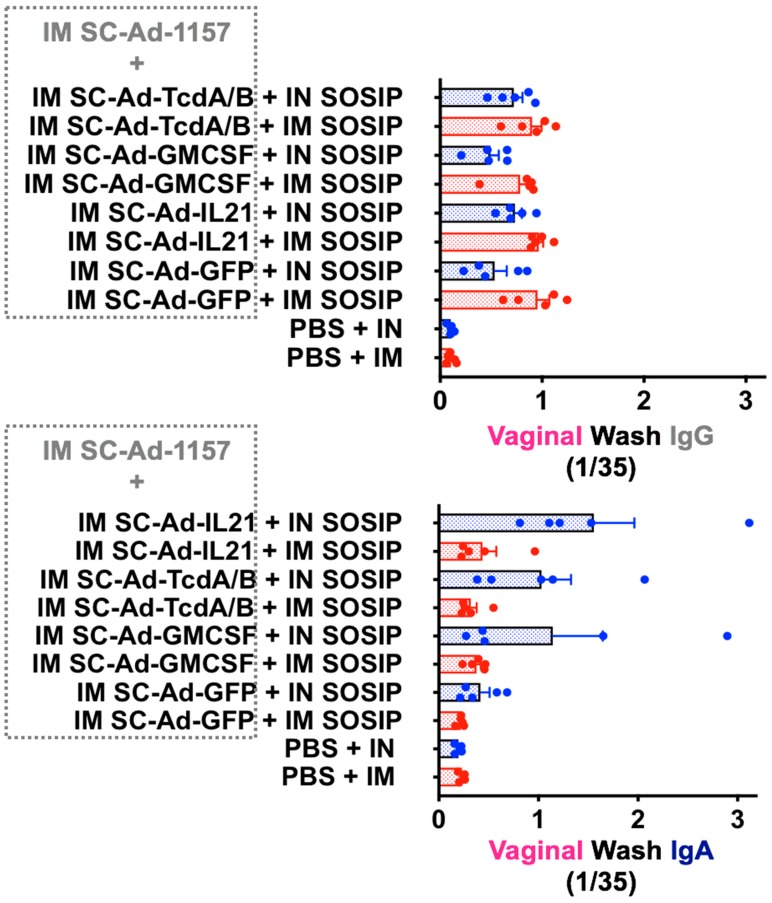
IM or IN SOSIP protein boost of SC-Ad-env + SC-Ad-genetic adjuvants. The groups of 10 mice from Figure 3 were divided and boosted with 5 µg CZA clade C SOSIP protein adjuvanted with 1 µg alphaGalCer by either the IM or IN route. Two weeks later, 1/35 dilutions of vaginal washes were assay for anti-CN54 IgG or IgA antibodies by ELISA.

Most HIV vaccines use protein boosts to maximize anti-env antibody responses, usually by IM injection [7,8,9,10]. We previously used trimeric gp140 as a protein boost in our clade B HIV vaccine studies in macaques [43]. Since then, improved stabilized trimeric envelope proteins have been developed by several groups. Given this, we used native-like soluble SOSIP proteins designed by Dr. John Moore [56,57,58] to boost the responses generated by SC-Ad in Figure 3. Each of these mice were boosted with 5 µg of clade C CZA97 SOSIP.v4.2-M6.IT produced from CHO cells [64] that was supplied by Dr. Moore and colleagues. The SOSIP protein was mixed with the NKT cell adjuvant alphaGalCer that we have used previously [59,60]. One half of the mice were boosted by the IM route, and one half were boosted by the IN route. Two weeks later, vaginal washes were collected and assayed for IgA or IgG antibodies against clade C env (Figure 4).

These data showed a strong bias in antibody responses based on the route of delivery of the SOSIP protein boost. IM SOSIP increased vaginal IgG levels generated by IM SC-Ad-1157 and SC-Ad GFP-Luc or GMCSF better than IN protein. In contrast, IN SOSIP protein boost strongly amplified vaginal IgA levels in mice that were primed by the IM route with SC-Ad-1157 with the strongest SC-Ad adjuvants: GMCSF, TcdA/B, and IL-21. The SC-Ad-1157 + SC-Ad-GFP-Luc group showed robust IgG responses when primed and boosted intramuscularly, but failed to generated a strong IgG response when the SOSIP was given IN. Furthermore, either of these combinations failed to generate IgA responses. This would suggest that genetic adjuvants that are given in place of SC-Ad-GFP-Luc prime the animals to drive the IgA responses we observe when they are boosted IN. Expectedly, the protein administered to unprimed animals generated little IgG or IgA response in vaginal washes.

## 4. Discussion

This study was performed to improve the ability of SC-Ad vaccines to generate mucosal immune responses. In particular, we sought to improve the generation of antibody responses at distant vaginal mucosal sites after immunization by clinically-relevant intranasal mucosal route of vaccination.

We showed that SC-Ad-expressing HIV clade B envelope combined with recombinant gp140 protein boosts could generate significant ADCC neutralizing antibodies against HIV-1 in nonhuman primates after IN or IM immunization [43]. IN immunization with SC-Ad appeared to generate better responses in plasma samples, but IN priming also generated mucosal antibodies only near the site of immunization and not at distant vaginal sites.

We show here in small animals that vaginal antibody production after IN immunization with SC-Ad can be markedly amplified by coadministration of SC-Ads expressing the genetic adjuvants GMCSF and TcdA/B. We also show that these genetic adjuvants along with IL-21 mediate improvements in systemic antibody responses after high dose SC-Ad IM immunization. Notably, under these conditions, IL-21 was most robust in amplifying antibodies in vaginal washes by this route.

These data suggest that genetic adjuvants can have value in improving systemic and mucosal immune responses when they are expressed in SC-Ad vectors. This study also tested how the route of protein boosting affects the character of antibody responses at mucosal sites. We show that intramuscular protein boost with clade C SOSIP protein generally increases IgG antibodies against HIV envelope in vaginal washes, whereas an intranasal protein boost generally increases IgA antibodies in vaginal washes.

Conventional wisdom is that secretory IgA mediates much of the protection by mucosal vaccines vs. mucosal pathogens [65,66]. However, the RV144 HIV vaccine trial suggested that high plasma IgA can associate with higher risks of HIV infection [67]. This higher risk may be due to IgA antibodies blocking the ability of more efficacious IgG isotypes from binding envelope. For example, IgG1 and IgG3 antibodies against V1V2 of env may correlate to protection with the RV144 vaccine [67]. While these IgA and IgG antibodies may have these potential biases, it is notable that the RV144 vaccine was not delivered by any mucosal route and that mucosal IgG and IgA responses were not assessed. It is therefore possible that the routes of immunization may have generated less effective IgA antibodies than might have been produced by a legitimate mucosal vaccination route.

Genetic adjuvants have usually been used by the IM route to amplify systemic immune responses, not to improve mucosal responses. In this study, SC-Ad vectors expressing a clade C envelope antigen were coimmunized with SC-Ads expressing 4-1BBL, GMCSF, IL-21, and a novel mucosal adjuvant that expresses the receptor-binding domains of *C. diff.* toxin A and B (TcdA/B) [45]. We tested if SC-Ads expressing genetic adjuvants could amplify mucosal responses and reduce the mucosal distance effect.

We will not know if these similar vaginal IgA and IgG responses can be driven in nonhuman primates with similar adjuvant genes until this is tested empirically. If similar biased responses can be provoked, this will allow an interesting head to head comparison of the utility of these isotypes that are generated by active immunization in protecting against mucosal SHIV challenge. Unlike passive immunization with purified IgA or IgG antibodies, these active gene-based vaccines also generate supportive T cell responses that influence the quality of these responses and also provide CD8 T cell backup to the antibodies. We anticipate protection mediated by mucosal IgA plus cellular responses versus mucosal IgG plus cellular responses may both generate potent protection if IgA can protective on its own and not obstruct protective IgG antibodies.

## 5. Conclusions

These data support the premise that the route of administration of SC-Ad vaccines and also the route of protein vaccine delivery influence the quality, quantity, and location of mucosal immune responses against HIV antigens. The findings of the present study suggests that SC-Ad genetic adjuvants may have ability to reduce the distance effect on mucosal antibody production when using clinically-relevant intranasal immunization. This also suggests that the character and isotype of mucosal antibody responses can be modulated by mucosal or systemic immunization with SC-Ad and protein vaccines. Together, these data may be useful in informing vaccine schedule designs to achieve specific immunological outcomes.
